# Improving Patient Satisfaction by Using Design Thinking: Patient Advocate Role in the Emergency Department

**DOI:** 10.7759/cureus.3872

**Published:** 2019-01-12

**Authors:** Solomon Feuerwerker, Nick Rankin, Brittany Wohler, Henry Gemino, Zachary Risler

**Affiliations:** 1 Medical Education, Sidney Kimmel Medical College at Thomas Jefferson University, Philadelphia, USA; 2 Emergency Medicine, Sidney Kimmel Medical College at Thomas Jefferson University, Philadelphia, USA

**Keywords:** design thinking, patient satisfaction, preclinical student, design, medical education, advocacy, patient advocacy, qi, quality improvement, emergency medicine

## Abstract

The emergency department (ED) serves a pivotal role in the healthcare system, but it is often a source of anxiety and confusion for patients at a time already shrouded by fear of illness and uncertainty. Common patient needs include receiving information about different stages of their care, assurance that they are safe, and knowledge of a plan for proper follow-up care prior to discharge. Due to well-known restraints on the clinician’s time, meeting this level of patient satisfaction has often fallen short. Design thinking is a well-known methodology used to generate solutions to a wide variety of problems with an approach that is inherently iterative in nature. The key feature of the process is a strong focus on practicing empathy as an approach to human-centered design. Utilizing this method, we created a role, filled by preclinical medical students, who are placed in the ED during peak hours to focus on making the patients more comfortable and tend to their more “non-clinical” needs. We posit that this new role will do the following: 1) make patients feel more satisfied with their care in the ED, 2) allow students to gain a robust appreciation for the flow of the ED and the hospital in general, and 3) teach students to actively solve patient's frustrations.

## Introduction

Emergency departments (EDs) provide treatment for a broad spectrum of illnesses and injuries with annual visits in the United States reaching 138 million [1]. Patients and their families will interact with a myriad of providers and support staff including attendings, residents, nurses, students, technicians, custodial staff, and general employees. They will be informed of tests and studies they may not understand while trying to drown out the loud noises emanating from machines and overhead speakers in an already overcrowded environment.

Satisfaction is known to be highest when patients feel that there is proper communication between them and ED providers [2-3]. Furthermore, patients often care less about the actual time spent waiting in the ED, so long as they are kept informed about wait times and next steps [4]. It is evident that EDs are in need of a more personal approach that engages and informs patients in addition to providing high-quality medical care.

There is no question that the ED serves a pivotal role in the healthcare system, but it is often a source of anxiety and confusion for patients at a time already shrouded by fear of illness and uncertainty. While clinical needs are of paramount importance, mitigating angst and ambiguity can serve to facilitate an environment where patients feel their needs are being met. Bridging this gap continues to pose a challenge in the already overstretched EDs. To accomplish this, we propose the creation of a new position in the ED called an ED patient advocate. This is a position filled by medical students, specifically designed to keep patients informed, while also serving as their advocate and as a familiar face in a foreign place. The intent of this report is to share how we applied the design-thinking process (defined below) to construct a feasible solution that serves to not only benefit the patient but also the medical students as they transition from the classroom to the wards. Furthermore, it is our hope that other institutions might adopt this solution, process, or both, in order to solve similar issues related to patient care.

## Technical report

As part of the Medicine + Design program at our institution, Sidney Kimmel Medical College at Thomas Jefferson University, our team (ED faculty and medical students) sought to identify a problem in the ED that could be addressed using design thinking. Design thinking is a well-known methodology used to generate solutions to a wide variety of problems that are poorly defined or unknown. Its use has been well established in the fields of architecture, engineering, and technology but more recently has been proven to generate user-oriented solutions to complex problems in healthcare [5-6]. Utilization of this method hinges on framing a problem in a human-centric way by first understanding another’s perspective. One such method was developed at the Stanford d.school and is broken down into five key parts: empathize, define, ideate, prototype, and test [7].

Process

The design process is inherently iterative in nature, with multiple iterations through the different components of the process until a working solution is found. The first step in this process is empathy, where one tries to understand the problem from another’s perspective [8]. We began by conducting interviews with our “end users,” defined as the individual or group of individuals with whom you choose to empathize in order to better understand their needs. In our case, the “end users” are ED patients. We immersed ourselves within the ED to observe and discuss the specific pain points (problems, concerns, or complaints) encountered during a typical visit at various times throughout the day. The goal here was not to identify one specific problem, but rather to broadly understand the experience from the end users’ perspective. Through this process, we spent many hours speaking with patients and providers. In total, four students each spent approximately 20 hours observing and interviewing patients, physicians, and nurses in the ED. This resulted in conversations with 40 patients, 11 physicians, and six nurses and the ED's patient relations manager who receives and handles all of the non-clinical complaints from patients. Our team specifically asked about pain points in specific areas where patient care is delayed or where patients feel lost or frustrated in the ED. We asked questions such as “if you could change one part of your visit what would it be?” or “at what point during your stay did you feel frustrated or confused about your care?”

The next step is to define an existing problem by synthesizing the information gathered. The goal of this stage is the development of a “how might we” statement -- a tool used in this method of design thinking to help narrow the focus of the problem in order to reach a stage where ideas can be generated in response to this framing of the problem. From speaking with patients and providers, we noticed a common thread of mismatched expectations, confusion about surroundings, and current work-up status; hence, we asked ourselves, “how might we better co-manage patient expectations in the ED?”

With a defined problem, end user, and focused question, we moved onto the third step: ideate. This is a stage of rapid prototyping, where numerous solutions are generated and tested. The technique we used was the brainstorm, in which the team of medical students and emergency physicians individually generated as many ideas as they could within a given 10-minute timeframe. Each of us wrote our ideas on post-it notes and stuck them on the board; the key at this step in the process is that no idea is too outlandish or impossible. This took place in our health design lab in parallel with four other teams of students and physicians, and at various stages of this process, the groups presented possible solutions for their defined problems in order to receive and give feedback. Applying this technique, our team came to the conclusion that the best solution would be to create a position, staffed by an individual familiar with the ED and hospital environment who would be tasked to help co-manage patient concerns and expectations to help navigate their ED stay.

We considered a variety of other possible solutions: posters detailing wait times, phone- or computer-based applications showing pending tests and kiosks explaining the ED operations. We realized that as technology has become more ubiquitous, people have become more frustrated with automated responses and impersonal forums. Additionally, it has been shown that the strongest predictors of patient satisfaction focused on “expressive quality” and interpersonal skills of ED providers, not technological updates [2,9]. A sign indicating average wait times pales in comparison to someone taking a moment to explain the details that are specific to a given patient. An app might be able to show in real time which tests are pending or complete but they lack the context and education that most patients need. Furthermore, although physicians in this setting have a remarkable ability to balance clinical evaluation and patient education, most would agree that they would like to spend more time with their patients for this very reason but are very often unable to because they are stretched too thin [10]. Thus, the team reached the solution as outlined above.

The next steps in the design process are to prototype and test, respectively. Traditionally, this involves building a mock-up of one’s idea for a simulated or real environment. In our case, we simultaneously tested and prototyped by having medical students try out the role of patient advocate in the ED. Students identified patients at triage, explained their role in the ED, communicated salient information to relevant staff members, and routed questions to the necessary providers to address their patients concerns. The specifics of their role and scope of practice are laid out in Appendix 1.

Solution

Our solution to address patient confusion was to create a new role in the ED. This would address the need for providing patients with a better understanding of the ED process. For example, how long they could expect to wait on a CT scan or what is going to happen to them after their blood work is completed. In addition, by providing a person that could empathize and listen to patients, our solution would directly address the inherent anxiety that patients face in the ED.

The role of a patient advocate would be filled by preclinical medical students who are both required and eager to get early clinical exposure in their training. In addition to serving patients needs, the preclinical medical students would have early opportunities to learn to navigate the clinical environment and the needs of patients. According to a systematic review by Dornan, early clinical exposure has shown significant positive outcomes for medical students including increased empathy towards patients and a more positive outlook on practicing medicine [11]. Thus, this solution fits well with the goals and objectives of the medical school curriculum.

The primary role of the ED patient advocate is to act as a unifying entity, bridging the gap between a busy ED staff and a confused patient. To help the advocate meet this goal, we created the following outline as a structure for how they might begin and facilitate conversations with patients:

**A** – Address. Address the patient, introduce yourself and ask how the patient would like to be addressed.

**E** – Empathize. “The emergency department can be hectic and confusing. Hopefully, I can help clarify things and address any concerns or questions you have while you’re here.”

**I** – Inform. Explain the layout of the ED, who is who and depending on your knowledge of the plan, what is going to happen next.

**O** – Offer Assistance. Ask if there is anything you can help with right now, specifically non-medical concerns.

**U** – “You statement.” For example, “You are waiting for X.” or “You are going for a CT scan.” Patients ultimately want to know what is happening now and what’s to come next.

With this structure, the patient advocate has an outline to bridge the gap between the providers and the patients. Ultimately, the role is to be present for patients and for the team and offer assistance whenever possible.

In addition to defining the role of the ED patient advocate, we delineated the logistics of the role and the “do’s and don’ts” in our ED Patient Advocate Program Guide (Appendix 1). This guide is an instruction manual for the preclinical medical students serving in the role and outlines the specifics of their role and scopes of practice.

## Discussion

Using the design process, we created an intervention that could improve patient care and satisfaction. This process allowed us to empathize with patients, then ideate, and finally test possible solutions. We are hoping this new process will help patients feel more comfortable with their ED experience as it will improve communication with the patient and the healthcare team.

The relationship between proper patient communication and ED satisfaction ratings is well established. In one study of ED patient satisfaction which included over 3,000 patients across 23 EDs, “expressive quality” and “information delivery” were two of the most statistically significant factors related to overall patient experience [2]. Expressive quality refers to interactions with staff and general bedside manner. Information delivery refers to patient’s satisfaction with how well they were informed about wait times, delays, and tests. In another study of over 2,300 ED patients assessing 68 potential determinants of satisfaction with patient care, five of the 10 strongest factors were also related to expressive quality and information delivery [4]. Patients reported that they were more concerned with being informed about their wait than the actual wait time itself. A review that examined the ED patient satisfaction literature and looked at over 50 independent studies determined that the most robust predictor of satisfaction is the interpersonal skill of the ED provider [9]. It is known that patients are more likely to return to the ED after discharge if there is perceived inability for follow-up care or fear of progression of disease, providing a unique opportunity for this curriculum to help assuage patient fears and ensure patient comfort with plan prior to discharge [12-13]. The evidence suggests that EDs are in need of a more personal approach that engages and informs patients.

Medical students can serve a unique role to engage and personalize the ED experience. This will serve the patients to improve their time in the ED as well as help the students as they transition to their clinical years. This transition can be challenging and studies have shown that students with early clinical experience have a more solid foundation of clinical knowledge and have also been shown to have more confidence when interacting with patients [11,14]. Furthermore, this exposure also has been shown to have a significant effect on students specialty choice [15]. The clear benefit of early clinical exposure during medical training has been well studied [16-17]. In a systematic analysis, which aggregated outcomes from 73 studies on the effects of early clinical exposure, significant positive outcomes were reported for students in both academic and professional domains [11]. They reported better communication skills, increased motivation, and a greater sense of identification with their profession during their early training. Students were also found to have a greater understanding of hospital infrastructure and were, therefore, better equipped to navigate the healthcare system. Academically, the early clinical exposure aided in students’ learning of biomedical and cognitive sciences.

The next steps for this project are already underway, with a pilot of preclinical medical students being trained to start in the role of ED advocate under the direction of EM faculty at our institution. Further research looking at patient perception of this new role is needed as the advocates interact with more patients, with possible studies of improvement of patient satisfaction in the future.

## Conclusions

The design process can be implemented in medicine to help with unmet needs with a strong focus on empathizing with the end user. In applying the design thinking methodology, we found a new and innovative solution to better serve our patients in their most vulnerable moments. One possible solution that we created using the design process is the ED patient advocate – preclinical medical students placed in the ED during peak hours to focus on making the patients more comfortable and tend to their more “non-clinical” needs. Students will gain a robust appreciation for the flow of the ED, the hospital in general as well as learn to solve problems with patients who are frustrated about their experiences.

## Figures and Tables

**Figure 1:**
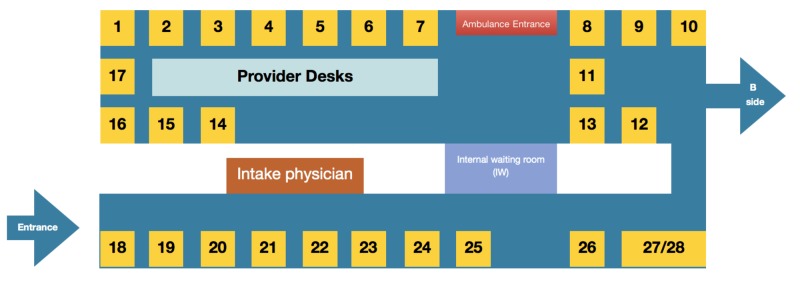
ED layout Numbered yellow boxes denote patient rooms in the ED ED: emergency department

## Appendices

Overview of advocate responsibilities

Day-to-day Breakdown

Prior to the start of shift: Dress nicely, we recommend business casual with your ED patient advocate shirt. Make sure to bring your Jefferson ID. Please do NOT wear your white coat as this may be confusing to the patient. Bring a writing utensil and something to write on, as it can be useful to take notes throughout the experience. Remember to be mindful of the Health Insurance Portability and Accountability Act (HIPAA) and to protect patient privacy at all times. All information written down during your shift must be disposed of in specially marked boxes specific for protected health information (PHI). If you are unsure of where to dispose of protected information, please ask the unit secretary. 

Arriving at the main entrance to the ED: The Thomas Jefferson University (TJU) ED is located on 111 S 11th St, Philadelphia, PA 19107. Please report to the attending physician working in intake (triage) at the start of your shift — if you have a partner, wait for them before beginning. Once your partner arrives (if you have one), enter the ED through the turnstiles to the right of the entrance next to the security desk (not at the metal detector). Introduce yourself to security at the entrance and briefly explain your purpose/role in the ED. Your hospital ID should grant you access to the ED. Turn left immediately after the turnstiles, walk past the elevators, and swipe your ID to enter through the double doors. Once you pass through the double doors, turn right and look for the intake attending. Introduce yourself as an ED patient advocate.

Start of your shift: The faculty and staff at Jefferson are friendly, but very busy. Be respectful of their time. Please arrive on time so that you may introduce yourself to the intake ED attending physicians, but be flexible if the attending physician is with patients. Make your introduction brief, as well as your rounds throughout the evening.

Getting started relies on communicating effectively with staff, making them aware of your role and responsibilities and identifying an effective starting point. Unfortunately, you will not have access to Epic, the hospital’s electronic medical record, although this will hopefully change in the future.

If you are unable to locate an attending by the entrance (intake physician — see below for layout), go to the A side of the ED, introduce yourself to the residents and ask if they know of any patients who could use your help. The second set of physician desks is past the internal waiting room; this is where the “A” side attending and residents sit. 

If you are assigned to be on the “B” side, you will pass the “A” side desks, turn right, and walk to the back of the ED. At the end of the hallway, turn left and directly in front of you will be a similar layout, with the “B” side attending physicians and residents.

If you are completing a shift with another student, one of you should introduce yourself to the attending or senior resident in your assigned area and ask if there are any patients who could use your help. The flow is outlined in Figure 1.

Remember that though residents, physicians, and nurses will have a posted reference sheet for their education purposes, which details your role in the ED, their knowledge of this is limited by their involvement in the program as well as by how busy the shift is.

During your shift: After identifying a starting point and familiarizing yourself with the room layout of the ED, you will begin to make “rounds”. Your goal is to identify five or so patients that you feel you can help and follow throughout your shift. These can either be patients you meet and follow from intake or patients you’ve met who are already roomed. Building a patient census for yourself will be a dynamic process. Because the environment of the ED changes on a moment to moment basis, your judgment, along with the guidance of the ED providers, will dictate how you build your census. 

Example:

A. You and your partner show up on a pretty typical, moderately busy day. You decide the best plan of action is to split up. You decide to meet new patients at intake and follow them through their ED stay. Your partner begins to see patients already roomed that are also in need of your service. 

B. You show up to your shift and see that the ED is actually pretty quiet. You make sure not to say the “q” word too loud and begin seeing patients in intake as there are not many already roomed patients in need of assistance. 

C. You and your partner show up and the ED is packed. The internal waiting room is filled and triage is a never-ending stream of patients. You decide your role in intake may be limited as patients are waiting up to seven hours for rooms and your efforts should be put towards the patients who are already roomed. 

Primary goal of your shift: Act as a unifying entity, bridging the gap between a busy ED and a confused patient by following this rough outline of conservation:

**A** - Address. Address the patient, introduce yourself (again), and ask how the patient would like to be addressed.

**E** - Empathize. “The emergency department can be hectic and confusing. Hopefully, I can help clarify things and address any concerns or questions you have while you’re here.”

**I** - Inform. Explain the layout of the ED, who is who and depending on your knowledge of the plan, what is going to happen next.

**O** - Offer assistance. Ask if there is anything you can help with right now, specifically non-medical concerns. If the patients have questions about their workup, simply state that you may not have the answers to their questions right away, but can track down their physician and get answers for them. NEVER make promises or give medical advice to the patient. Any conversations about their care, workup or next steps should be well thought out and under the assistance or guidance of the providers as well as confidential. Make sure if there is anyone else in the room that they are okay with you speaking in front of them as well.

If patients have questions, when they arise, notify physicians. They may speak directly to the patient or will provide you with key information to convey back to the patients. The information you are to convey between patients and providers may include appropriate test/procedure information, updates regarding admission status, inquiries about comfort/pain, or questions regarding ED logistics. All of this information is protected information and not to be discussed with anyone outside the direct treatment team.

**U** - “Your statement.” IE. You are waiting for X. You are going for a CT scan. Patients ultimately want to know what is happening now or what’s to come next. Once you have firmly established what the next steps are, be definitive and clear about their work up or next steps. 

Ideally, the flow would go as follows:

Meet as many patients as you can in the intake area with the intake provider. Identify who would not only benefit from your care but also agrees to have you part of their treatment team. Once you feel you have met enough patients, you can leave intake to serve the patients you have identified. If you are unsure as to the where they were roomed, please speak to the providers to assist you in locating them. 

Begin your rounds. Check in on these patients to see how their visit is progressing and assist them as you are able (see above for outlined conversation). If you feel you could take on more patients, feel free to return to intake to meet new patients, or ask providers (attendings, residents, physician assistant/nurse practitioners, and nurses) if there are any additional patients that could use your help. We hope that you will be a friendly, comforting face for patients and their families during this stressful time!

Please remember: All medical students serving as ED advocates will have completed HIPAA training and therefore must abide by HIPAA regulations at all times. When relaying patient information to family members, you will need to be respectful of your surroundings and convey information only in private. You cannot share information with family members unless the patients have specifically given you permission to do so. Be especially careful with patient privacy if you are talking to patients in the internal waiting room, as there are often other patients nearby so you may need to adjust conversations accordingly. It is crucial NOT to overstep the boundaries of the ED while acting as an ED patient advocate. The role of the ED patient advocate is not clinical in nature. You will not obtain histories, perform physical examinations, or perform procedures. You will not personally interpret test results or imaging studies. You should only relay information provided by the ED attending to the patient. You should not look up test result information or imaging studies on the computers in the ED by yourself. These computers are reserved for those performing clinical duties in the ED. You will be a friendly face and should be a comfort to the patient. As such, the patient may ask your opinion. It is important that you do not offer prognosis or treatment recommendations to the patient. It is also not your role to order any tests, or perform estimates regarding a timeline of when a patient can expect a result or treatment unless you are specifically instructed to do so by your attending. Table 1 provides a helpful summary of the "do's and don'ts" for this role. 

**Table 1:** Tips for advocates

DO	DON'T
Bring a positive attitude and a smile on your face	Give medical advice of any kind (except if relaying information from the provider)
Introduce yourself often! (make sure the providers and patients in the ED know who you are and why you are there)	Give a prognosis
Wear your ED patient advocate shirt	Give time estimates (unless specifically told to do so by a provider)
Inform patients and their families that you are there to make them as comfortable as possible	Take a history or perform a physical exam (you may ask specific questions if a provider needs your help collecting information)
Take initiative, seek out patients to talk to and make it known to the ED staff that you are ready to help in any way	Perform any medical procedures (even if asked by the resident or attending!)
Be a team player, remember you are here to help the patients and staff	Discuss ANY patients you have seen or their medical information outside of the hospital/people taking care of them (this is a HIPAA violation)
Learn: about patients, about the ED, about the hospital, about medicine and about yourself!	Leave the ED with ANY identifiable patient information, written or printed
	Discharge a patient on your own

End of shift: Make sure to let your patients know you will be leaving and ask them if they have any final questions or concerns that you should convey to the attending physician before your leave. Also, remember to thank the ED attending physicians and staff you have been working with. If patient advocates are working after your shift brief them on your patients, as sign-out and continuity of care is an invaluable part of working on a medical team.

Sign out:

1. Room numbers you are following

2. Current status of their workup and established plan

3. The patient’s primary concerns/needs while they have been in the ED

If you are receiving sign-out and feel that you can see more patients, please return to the intake area to meet additional patients.

Remember: On busy days, seeing patients in intake may be challenging. Even if you are able to see them, they may be waiting in the internal waiting area for hours (sometimes up to seven hours!). Therefore, at particularly busy times, it may be more beneficial to speak with providers about adding patients to your census that are already roomed who may benefit from your care. Though ideally, patient advocates would be a familiar face from start to finish, in reality, the chaos of the ED may not cater to this idea and remember that everyone in the ED could use your help in some way!

If something goes wrong

Medical

If a patient seems to be in emergent distress, do NOT try to do anything by yourself, call for help immediately!

If serious but not emergent/life-threatening, please seek out the resident or nurse taking care of the patient to let them know that they are needed.

Formal Patient Complaints

Mr. A is the patient advocate in the ED and handles all of the complaints related to care received in the ED. He is incredibly friendly, knows about your role in the ED, and has offered to always support you as a student if something comes up during your shift. If it is during business hours, it may be helpful to have him come and have a conversation with the patient, you can do this by calling him at 123-456-7890. If not, provide the patient/family member with his information and send him an email () with a quick few sentence description of what might have happened so he may handle it from there. Remember, the goal is not to simply pass off every issue to Mr. A - involving him is a pathway for when you feel escalation is necessary.

Program/Student Related

Contact B, the Education Program Administrator for the JeffMD Clinical Experience Program:
